# Outcomes in Ovarian Cancer among Hispanic Women Living in the United States: A Population-Based Analysis

**DOI:** 10.1155/2013/672710

**Published:** 2013-02-20

**Authors:** Okechukwu A. Ibeanu, Teresa P. Díaz-Montes

**Affiliations:** Division of Gynecologic Oncology, Department of Gynecology and Obstetrics, The Kelly Gynecologic Oncology Service, The Johns Hopkins Medical Institutions, Baltimore, MD 21287, USA

## Abstract

*Introduction*. Ovarian cancer is the deadliest gynecologic cancer in the United States. There is limited data on presentation and outcomes among Hispanic women with ovarian cancer. *Objective*. To investigate how ovarian cancer presents among Hispanic women in the USA and to analyze differences in presentation, staging, and survival between Hispanic and non-Hispanic women with ovarian cancer. *Methods*. Data from January 1, 2000 to December 31, 2004 were extracted from the National Cancer Institute's Surveillance, Epidemiology and End Results (SEER) database. *Results*. The study sample comprised 1215 Hispanics (10%), 10 652 non-Hispanic whites (83%), and 905 non-Hispanic blacks (7%). Hispanic women were diagnosed with ovarian cancer at a younger age and earlier stage when compared to non-Hispanic whites, non-Hispanic blacks; *P* < 0.001. Similar proportion of Hispanics (33%), non-Hispanic whites (32%), and non-Hispanic blacks (24%) underwent lymphadenectomy; *P* < 0.001. Hispanics with epithelial ovarian cancer histology had longer five-year survival of 30.6 months compared to non-Hispanic whites (22.8 months) and non-Hispanic blacks (23.3 months); P  =  0.001. *Conclusion*. Hispanic women with ovarian cancer have a statistically significantly longer median survival compared to whites and blacks. This survival difference was most apparent in patients with epithelial cancers and patients with stage IV disease.

## 1. Introduction

Ovarian cancer is the leading cause of mortality among gynecologic cancers diagnosed in the United States (USA) [1]. The American Cancer Society has estimated that approximately 22,280 new cases of ovarian cancer will be diagnosed during 2012 making it the ninth most common cancer diagnosed among women living in the USA. It is also estimated that 15,500 women will die of this disease, making it the fifth most common cause of cancer-related deaths among women in the USA during 2012 [2]. Sporadic ovarian cancer has a peak age of incidence of 60–70 years [1]. The disease is typically diagnosed in advanced stage (stage III/IV), partly because there is no routine screening procedure for ovarian cancer among women in the general population [3], and as yet, no reliable screening biomarker has been detected. Cancer Antigen (CA 125) measurements do not have adequate sensitivity and specificity to satisfy requirements for use as a screening tool [4]. Overall 5-year survival rate in women with ovarian cancer is 46% and is related to stage of disease at diagnosis. Five-year survival rates are 93%, 71%, and 31% for local, regional, and distant disease, respectively [5]. Important prognostic factors for ovarian cancer are age, histology (serous versus nonserous), stage of disease, and the volume of residual disease after cytoreductive surgery [3]. Current standard treatment is primary cytoreductive surgery and adjuvant platinum-based chemotherapy.

Ovarian cancer trends have previously been evaluated among several ethnic groups in the USA. A review of the Surveillance, Epidemiology, and End Results (SEER) data from 2005 showed that Caucasian women had the highest incidence rate of ovarian cancer (12.9 per 100,000 population) followed by Hispanics (10.9 per 100,000 population), African-Americans (9.3 per 100,000 population), Asian/Pacific Islander (8.7 per 100,000 population), and American Indian/Alaska Natives (7.0 per 100,000 population) [6]. Caucasian women also had the highest mortality rates (9.0 per 100,000 population) followed by African-Americans (7.2 per 100,000 population), Hispanics, and American Indian/Alaska Natives (6.0 per 100,000 population) and Asian/Pacific Islander (5.0 per 100,000 population) [6].

Hispanics currently make up the largest and fastest-growing minority ethnic group in the USA, constituting 12.5% of the population and are expected to make up one quarter of the total population by 2050 [7]. At present, there is a paucity of data regarding age at diagnosis, histology, stage of disease, grading, treatment offered, and prognosis among Hispanic women diagnosed with ovarian cancer. Given the huge healthcare costs in the USA, and the expected increase in cancer cases diagnosed in the coming decades, it is important to have accurate knowledge of the distribution of disease, disparities in risk factor prevalence, treatments, and outcomes among various subgroups of the population, in order to use healthcare resources efficiently.

The goals of this study are to investigate how ovarian cancer presents among Hispanic women living in the USA and to evaluate the differences in presentation, staging, and survival between Hispanic and non-Hispanic women diagnosed with ovarian cancer.

## 2. Methods

Demographic, clinico-pathological, treatment and survival information from women with ovarian cancer from January 1, 2000 to December 31, 2004 was extracted from the National Cancer Institute's Surveillance, Epidemiology and End Results (SEER) Program database. The SEER Program is an authoritative source of information on cancer incidence and survival in the USA, which collects information and publishes cancer incidence and survival from 12 population-based cancer registries and 3 supplemental registries that encompass nearly 14% of the total USA population [8]. Included in the analysis were all women identified as Hispanic, Non-Hispanic black, and non-Hispanic white under the race coding with a diagnosis of ovarian cancer under the primary site coding for cancer (183.0 ICD-O) [9]. Any cases that did not meet all of the above criteria were excluded from our analysis. The study was exempt from Institutional Review Board (IRB) approval because the information obtained is not individually identifiable and was not collected by direct contact with participants specifically for this study. The SEER Program database is of public use and is federally funded by the National Cancer Institute.

Statistical analysis was performed using the SPSS version 17.0 statistical software program (SPSS Inc, Chicago, IL, USA). Descriptive statistics were used to present demographic data. Chi-square test was used to analyze the differences between the groups with respect to demographic variables, disease and treatment characteristics. Kaplan-Meier survival analysis was performed and log-rank tests were used to compare survival among racial groups. Cox regression method was used to analyze independent factors that were related to survival. A two-sided *P* value < 0.05 was considered statistically significant.

## 3. Results

A total of 12,772 women were included in the study. Overall, the mean age at diagnosis among the cohort of women was 62.6 years. Hispanic women were diagnosed with ovarian cancer at a younger age (54.5 years) when compared to the non-Hispanic groups (non-Hispanic whites: 63.8 years, non-Hispanic blacks: 59.8 years; *P* < 0.001). Sixty-nine percent of the women in the cohort were 55 years of age or older. Whereas 51% of the ovarian cancer cases among Hispanic women presented at age 55 years or younger, only 28% of non-Hispanic white and 37% of non-Hispanic black women were in this age group (*P* < 0.001) (Table 1). Hispanic women also had a lower frequency of ovarian cancer being diagnosed at 65 years and older when compared to the non-Hispanic groups (Hispanics: 32%, non-Hispanic whites: 50%, and non-Hispanic blacks: 43%; *P* < 0.001) (Table 1).

Fifty-three percent of the women in the cohort were diagnosed with advanced stage (III-IV) disease and 26% with early stage (I-II) at the time of initial presentation. Hispanic women had a higher proportion of ovarian cancer being diagnosed at an earlier stage (I-II) when compared to the non-Hispanic groups (Hispanics: 32%, non-Hispanic whites: 26% and non-Hispanic blacks: 26%, *P* < 0.001). The majority of the women (60%) in the cohort were diagnosed with epithelial ovarian cancer. This trend was noted among the different ethnic groups (Hispanics: 57%, non-Hispanic whites: 61%, and non-Hispanic blacks: 49%) (Table 1).

Only 31% of the overall study population underwent a lymphadenectomy during the initial surgical staging. Similar proportion of Hispanics and non-Hispanic whites underwent a lymphadenectomy, as compared to non-Hispanic blacks (Hispanics: 33%, non-Hispanic-whites: 32%, and non-Hispanic blacks: 24%) (*P* < 0.001) (Table 1).

The overall median survival of the study population was 36 months. Hispanics had a median survival of 45 months, which was statistically significantly longer when compared to 36 months for non-Hispanic whites and 24 months for non-Hispanic blacks (*P* < 0.001). As expected, age at diagnosis of 54 years or younger, early stage (I-II) disease, low-grade histology, and negative lymph node status were associated with longer median survival across all groups (Table 2). Hispanics with an epithelial histology had a survival of 30.6 months which was longer when compared to non-Hispanic whites (22.8 months) and non-Hispanic blacks (23.3 months) (*P* = 0.001) (Table 2).

**Figure 1:**
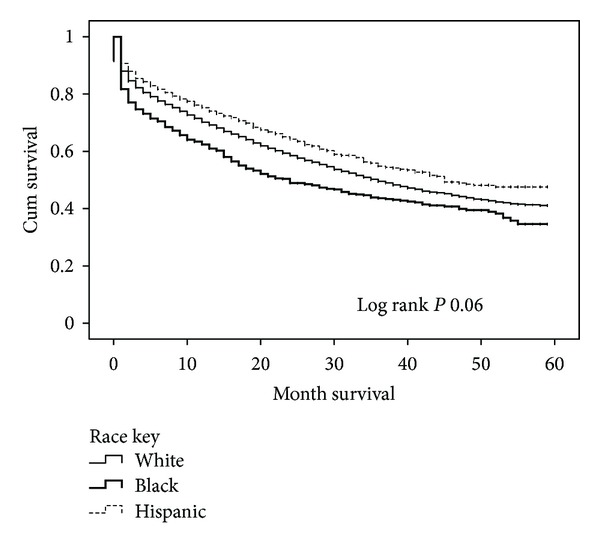
Kaplan-Meier survival curve of all patients based on ethnicity.

Kaplan-Meier survival curves are shown in Figure 1, illustrating 5-year survival values for overall survival and median survival for Hispanic patients. The differences were not statistically significant.

## 4. Discussion

In 2005, immigrants represented twelve percent of the total USA population. Fifty-three percent of all foreign-born individuals in the USA were from Latin America [10]. Hispanics currently make up the largest minority ethnic group in the United States and are expected to grow to one-fourth of the USA population by 2050 [7]. Unfortunately, there are few studies reporting on the outcomes of gynecologic malignancies among Hispanics. In addition, the demographic factors that influence the presentation, management, and prognosis among Hispanic women with ovarian cancer remain under-reported.

On average, Hispanics were diagnosed at an age 9.3 years younger than non-Hispanic whites and 5.3 years earlier than non-Hispanic blacks. These findings may be related to the larger proportion of younger age group individuals in the Hispanic subpopulation, according to USA census data [3]. It is also observed that the incidence of epithelial ovarian cancers is lower among Hispanics than Caucasian women [3]. The lower incidence rate of epithelial tumors could be partly linked to the fact that Hispanic women have higher numbers of offspring and practice breastfeeding more commonly than Caucasian women; both of which are protective factors against the development of ovarian cancer [11].

Uncharacterized genetic factors could also be involved in ovarian carcinogenesis among this Hispanic cohort. Ovarian cancer occurs at a younger age among BRCA mutation carriers. Some investigators have reported on hereditary ovarian cancer-related genetic mutations among certain clusters of Hispanic women showing that BRCA 1/2 mutations exist among Hispanic probands in the United States and Latin America [12–14].

More Hispanic women in this analysis had early stage disease (32.2%) than Caucasians (26.2%) and blacks (25.9%). This difference was statistically significant. The reasons for this are not clear; however, one clinically relevant consequence is that earlier age at presentation among Hispanics may have influenced the use of aggressive primary surgical treatments with the expectation of lower surgery-related morbidity in younger aged patients. Further analysis of the cohort shows that a statistically significantly larger proportion of Hispanic women underwent staging lymphadenectomy as part of their treatment. The prognostic effect of lymphadenectomy in early stage ovarian cancer is well known [15]. The higher frequency of lymphadenectomy in Hispanics is unexplained but suggests more frequent use of complete staging procedures among Hispanics as a proxy measure of health process measure. Process-related measures and outcomes merit further study in this group of women.

Overall, our data indicate that Hispanics with ovarian cancer have a statistically significantly longer median survival compared to whites and blacks. This survival difference was most apparent in the patients with epithelial cancers and those with stage IV disease. Stage for stage, Hispanic patients had a more favorable survival profile than the rest of the cohort. The survival difference was not statistically significant.

The current study has several limitations that must be considered when interpreting the results. First, the use of the SEER database as our source of patients lacks external validity. The SEER database only represents 14% of the USA population. The configuration of the SEER data set does not allow stratification of different Hispanic subgroups in order to evaluate whether the diagnosis of ovarian cancer at a younger age is a Hispanic characteristic or whether it is more strongly related to the subethnicity within the Hispanic community. There is incomplete followup, and also, the self-reporting of race and ethnicity may be subjective. The information in the SEER database does not provide information on provider specialization.

Despite these limitations, the current study provides the most extensive comparison of ovarian cancer patterns between Hispanic and non-Hispanic women in the United States. The observation that Hispanic women are diagnosed with ovarian cancer at an earlier age than non-Hispanic groups underscores the need for additional studies to explore this trend. The information obtained from this study is useful given the anticipated changes in the USA population over the next few decades. This could influence the allocation of health care resources and guide the provision of specialized cancer care to at-risk demographic groups.

## Figures and Tables

**Table 1:** Age at diagnosis, histology, and extent of disease among Hispanic, non-Hispanics white, and non-Hispanics black women diagnosed with ovarian cancer, US SEER Data 2000–2004.

Characteristics	Non-Hispanic white	Non-Hispanic black	Hispanic	Overall	*P* value
Age categories	*n* (%)				
<23 years 24–54 years 55–64 years 65–74 years >74 years	125 (1.2) 2860 (26.8) 2329 (21.9) 2313 (21.7) 3025 (28.4)	29 (3.2) 308 (34) 183 (20.2) 194 (21.4) 191 (21.9)	68 (5.6) 555 (45.7) 208 (17.1) 207 (17.0) 177 (14.6)	222 (1.7) 3723 (29.1) 2720 (21.3) 2714 (21.2) 3393 (26.6)	<0.001

Histology					
Epithelial Nonepithelial Other	6483 (60.9) 333 (3.1) 3836 (36.0)	440 (48.6) 46 (5.1) 4.9 (46.3)	693 (57) 111 (9.2) 411 (33.8)	7616 (59.6) 490 (3.9) 4666 (36.5)	<0.001

Stage					
I II III IV Unknown	2059 (19.3) 735 (6.9) 2873 (27.0) 2575 (24.4) 2390 (22.4)	169 (18.9) 63 (7.0) 208 (23.0) 263 (29.1) 202 (22.3)	308 (25.3) 83 (6.8) 311 (25.6) 253 (20.8) 260 (21.4)	2536 (19.4) 881 (6.9) 3392 (28.6) 3111 (24.4) 2852 (22.3)	<0.001

**Table 2:** Showing factors related to survival at 5 years among Hispanic, non-Hispanics white, and non-Hispanics black women diagnosed with ovarian cancer, US SEER Data 2000–2004.

Characteristics	Non-Hispanic white survival (months)	Non-Hispanic black survival (months)	Hispanic survival (months)	Overall survival (months)	*P* value
Median	36	24	45	36	<0.001

Age at diagnosis (years)					
<54 55–64 65–74 >74	47.4 39.2 32.3 18.4	42.8 29.8 24.6 12.0	46.6 36.2 28.8 12.8	46.9 38.4 31.5 17.9	

Stage					
I-II III-IV	52.2 29.1	51.0 23.0	52.7 30.5	52.0 28.7	<0.001

Grade					
1 2 3 unclassified	50.8 43.0 35.2 26.6	47.1 34.8 30.2 32.3	51.0 40.2 35.1 34.3	50.7 42.3 34.9 34.2	0.02

Histology					
Epithelial Nonepithelial	22.8 41.3	23.3 37.1	30.6 42.0	23.6 41.2	0.001
